# BIDScoin: A User-Friendly Application to Convert Source Data to Brain Imaging Data Structure

**DOI:** 10.3389/fninf.2021.770608

**Published:** 2022-01-13

**Authors:** Marcel Peter Zwiers, Stefano Moia, Robert Oostenveld

**Affiliations:** ^1^Donders Institute for Brain, Cognition and Behaviour, Radboud University, Nijmegen, Netherlands; ^2^Basque Center on Cognition, Brain and Language, San Sebastian, Spain; ^3^NatMEG, Karolinska Institutet, Stockholm, Sweden

**Keywords:** BIDS, GUI, conversion, neuroimaging, data sharing, open-source software, Python, plugin

## Abstract

Analyses of brain function and anatomy using shared neuroimaging data is an important development, and have acquired the potential to be scaled up with the specification of a new Brain Imaging Data Structure (BIDS) standard. To date, a variety of software tools help researchers in converting their source data to BIDS but often require programming skills or are tailored to specific institutes, data sets, or data formats. In this paper, we introduce BIDScoin, a cross-platform, flexible, and user-friendly converter that provides a graphical user interface (GUI) to help users finding their way in BIDS standard. BIDScoin does not require programming skills to be set up and used and supports plugins to extend their functionality. In this paper, we show its design and demonstrate how it can be applied to a downloadable tutorial data set. BIDScoin is distributed as free and open-source software to foster the community-driven effort to promote and facilitate the use of BIDS standard.

## Introduction

In the last few decades, neuroimaging data have become an increasingly rich source of information for studying the working of the brain in health and disease. Typically, the acquisition of such data sets is expensive and often difficult to collect from a large number of participants. Contemporary neuroscientific and clinical research questions are based on ever advancing analysis methods that require the availability of data sets that are very large (also known as “big data”) or of superior quality, or both. Several initiatives have been undertaken to address this problem by pooling the data from individual studies across the globe and by sharing data in online repositories (see, e.g., Turner et al., 2016, for a special issue overview).

The initial lack of data-structure standardization, data-sharing tools, and data-sharing mindset (Poline et al., 2012; Nichols et al., 2017; White et al., 2020) have led to the use of a large variety of file formats and data management methods, and to the lack of metadata descriptions, leaving researchers with the daunting task of adapting all the collected data in a custom format to run their analysis pipelines. Recently, the Brain Imaging Data Structure (BIDS; Gorgolewski et al., 2016) was introduced to alleviate this task to increase data sharing and usage and to facilitate reproducibility studies.

In essence, BIDS is a specification that prescribes how data collections should be organized and formatted on disk, in a computer and human readable way: it specifies the folder structure, the file names, the metadata fields, and its file formats. The BIDS standard was initially developed for MRI data but has since been embraced by the wider neuroimaging community, as indicated by extensions of the standard to MEG, EEG, iEEG, genetic, and PET data (Niso et al., 2018; Holdgraf et al., 2019; Pernet et al., 2019; Knudsen et al., 2020; Moreau et al., 2020), by a large number of BIDS Extension Proposals,^1^ a surge in BIDS apps (Gorgolewski et al., 2017), and a wide adoption of BIDS being used in publications. BIDS standard has moved the burden of homogenizing the data from the end user to the researchers who have collected the data set – and, importantly, who have the best knowledge about that data. In addition, the development of BIDS apps^2^ has provided researchers with easy to use, standardized processing pipelines that are typically well tested and documented.

Nevertheless, a limiting factor in the adoption of BIDS standard is that many of the neuroscientists who collect data do not have the programming skills to reformat their data in an efficient or automated manner. Various BIDS conversion command-line tools^3^ to support researchers have been made available, ranging from institute- or study-specific solutions, to community developed software, and from poorly documented tools to more advanced packages with programmatic interfaces. Among these, many use the well-known dcm2niix converter (Li et al., 2016) under the hood to perform the actual data conversion, such as the popular HeuDiConv (Halchenko et al., 2020), dcm2bids,^4^ and the related bidskit^5^ tools. HeuDiConv is a powerful tool but requires Python programming skills, albeit basic, and its rule-base heuristics design has a relatively steep learning curve and requires technical knowledge about the data. Dcm2bids uses a mapping approach that is easier to use although users still need to manually write their own configuration files. Solutions also exist for non-MRI data such as EEG or MEG data converters such as MNE-BIDS (Appelhoff et al., 2019), FieldTrip,^6^ and EEGLAB^7^.

A common limitation of the available tools is that they generally lack graphical user interfaces (GUIs) that can lower the barrier to adopt BIDS standard. To our knowledge, only a few converters come with a GUI. A service named as ezBIDS^8^ allows researchers to use a web browser to upload their DICOM data to a web server, to configure the dcm2niix-based data conversion, and to download a converted BIDS data set. Furthermore, a plugin^9^ for the Horos/OsiriX DICOM viewer uses dcm2niix to convert DICOM data to BIDS. Finally, pyBIDSconv,^10^ is an MRI-centered wrapper around dcm2niix, DataLad-hirni^11^ is an extension for DataLad (Halchenko et al., 2021), and Biscuit^12^ is an MEG-centered wrapper around MNE-BIDS. However, these three converters no longer seem to be under active development and do not support recent extensions to BIDS standard.

With the BIDScoin application suite presented in this paper, we aim to further promote the usage of BIDS by providing a flexible framework to convert any kind of source data to BIDS in a user-friendly way, which requires no previous programming knowledge. To achieve this goal, BIDScoin uses an intelligent mapping approach to associate raw source data types with BIDS target data types. The approach exploits as much of the digitally available information about the data as possible as well as the information that is typically known only by the researcher. The mapping approach of BIDScoin is intuitive for neuroimaging researchers as (1) it resembles the way they often think about their data types (they can recognize the data types they have collected when they see them, but do not know how to uniquely and reliably identify them technically), (2) it is simple and flexible as a virtually unlimited number of concurrent mappings can be established, and (3) it offers a GUI for users to directly and easily edit the mappings to their needs.

## Method

All BIDScoin codes are freely available at github^13^ and pypi,^14^ and the documentation can be found on Read the Docs.^15^ The latest BIDScoin version 3.7 as described in this paper is written in Python 3.6 and dependent on the freely available PyQt5 (Riverbank Computing Limited, Dorchester, England) software library for the GUI.

### The BIDScoin Workflow

**FIGURE 1:**
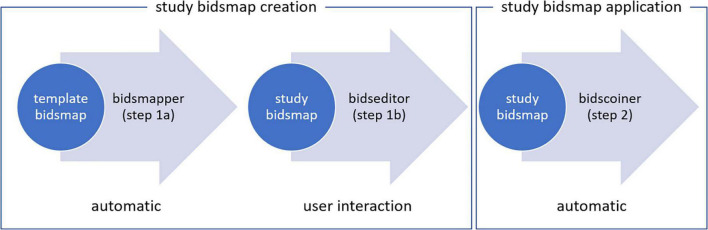
Creation and application of a study bidsmap. The user runs the “bidsmapper” executable with a “template bidsmap” as an input and with a “study bidsmap” as an output with the suggested Brain Imaging Data Structure (BIDS) data types, entities, and metadata. The study bidsmap is verified and edited interactively with the “bidseditor” graphical user interface (GUI). Finally, the study bidsmap is passed to the “bidscoiner” to convert the source data to BIDS.

**FIGURE 2:**
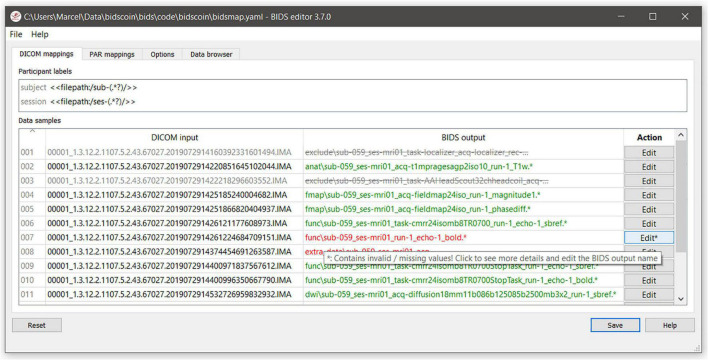
The bidseditor main window with an overview of the data types in the source data (left column) with a preview of the BIDS output names (right column). The green or red color indicates whether manual editing of the BIDS mapping is necessary, while the strikeout text indicates that the data type will not be converted, which is useful for handling irrelevant data. The user can edit the “subject” and “session” property values if needed (“session” can be left empty to be omitted) and the result is immediately reflected in the preview. Different tabs represent different data formats in the source data set, i.e., DICOM and PAR, which are represented as separate sections in the bidsmap. In addition, there is a tab to edit the study-specific “Options” and a tab in which the user can browse the organization of the source data and inspection of the data.

**FIGURE 3:**
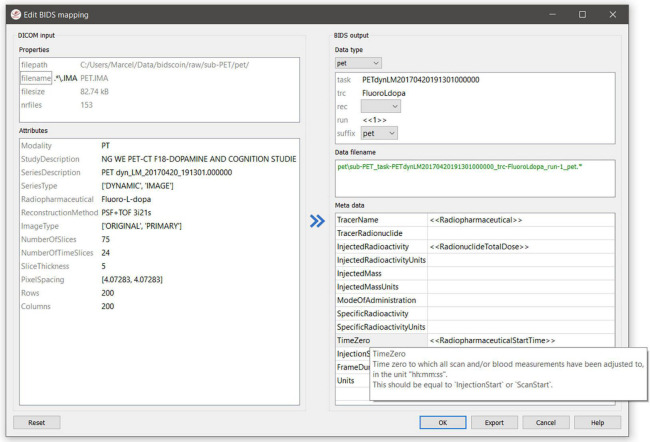
The BIDS mapping edit window featuring filename matching (.*\.IMA) and dynamic metadata values (e.g., “TimeZero”). The BIDS values that are restricted to a limited set are presented with a drop-down menu (here the “Data type,” the “rec,” and the “suffix” value). The user can immediately see the results of their edits in the preview of the BIDS output file name. A green file name indicates that the name is compliant with BIDS standard, whereas a red name indicates that the user still needs to fill out one or more compulsory BIDS values (with a pop-up window appearing if the user ignores it). Hoovering with the mouse over dictionary keys pops up explanatory text from the BIDS schema files, as highlighted for “TimeZero”. Double clicking on the DICOM file name opens a new window displaying the full header information with all attributes. The user can export the customized mapping to a different bidsmap on disk.

The workflow of BIDScoin to convert source data into BIDS standard consists of three steps (Figure 1):

(1a) To start with, the researcher runs a command-line application named as “bidsmapper” to perform the data discovery on their source data set (i.e., the folder containing all the input files). In this step, a so-called “template bidsmap” is used to scan the entire source data set and automatically create what will be referred to as a “study bidsmap.” Conceptually, the template bidsmap can be thought of as a set of broad filters, each of which maps a source data type onto a single BIDS output data type (e.g., anat, func, fmap, and dwi), onto an “exclude” data type that is not converted to BIDS, or, if none of the filters match, onto an unknown “extra_data” data type. Whenever a template filter matches with a source data type, the bidsmapper narrows the filter to exactly match to this particular source data type only and adds it to the study bidsmap if not present there yet. In this way, a mapping shortlist is built up in the study bidsmap, representing all of the unique source data types that are present in the source data folder. Note that a single broad filter from the template bidsmap can result in multiple narrow filters in the study bidsmap, for instance when two similar anatomical MRI scans are collected with different spatial resolutions. In the rest of this paper, we will refer to a bidsmap filter that maps to a BIDS data type (including the output file names and metadata) as a “BIDS mapping.” A template bidsmap is generic and typically created once, whereas a study bidsmap is tailored to the data at hand and therefore stored together with the output data.
(1b) After the study bidsmap has been created, a GUI application named as “bidseditor” is launched, either automatically by the bidsmapper or manually. The bidseditor reads in the study bidsmap and opens a main window that shows the shortlist of the discovered source data types and their suggested mappings to BIDS (Figure 2). From the main window, researchers can open subwindows to enrich or correct each of the suggested mappings using the knowledge they have about the data (Figure 3). In BIDScoin, prior (e.g., research center-specific) knowledge about the data can be represented in the template bidsmap: the more of this knowledge is represented, the larger the number of correctly suggested mappings will be, and the lesser edits the researcher needs to make. When the template bidsmap is unsuited or lacks any prior intelligence, all source data types will be classified as “extra_data” and the researcher will have to edit each mapping to the correct BIDS data type. Still, in such a worst-case scenario, the researcher has to perform only a limited amount of work on a short list of items. The bidsmap and all the user edits are immediately validated against the public BIDS schema files to ensure the specification of all mandatory fields and produce the correct metadata and valid naming of all the output files.

(2) After the data discovery and editing are done, the final step in the workflow is to call the “bidscoiner” application to automatically convert (“coin”) the source data set to a BIDS data set, as specified by the mappings in the study bidsmap. Note that as the number of mappings is independent from the number of subjects or sessions, the bidscoiner can be re-run every time new subects or sessions are added to the source dataset, without the need to re-run the bidsmapper or editor. If new scan protocols are employed for subsequent data, the researcher can repeat steps 1a and 1b first, which will reload the previously edited mappings and add the mappings for the new data samples to the list.

### The Bidsmap

#### Brain Imaging Data Structure Mapping

Thus far, we have referred to the bidsmap as a collection of BIDS mappings (filters) that define how the different source data types should be converted to the BIDS output data. Source data typically comes with two sources of information about the datatype and acquisition parameters, namely (1) metadata that is inherent to the filesystem, such as parts of the folder or file name, and (2) metadata that is intrinsic to the data itself, such as information represented in the header of the binary file. Depending on the imaging modality and on the data management plan, researchers often use either one of these sources or both. For instance, as opposed to MRI data in the DICOM format, most EEG data formats contain rather limited header information. Similarly, in the BIDS format, metadata is also stored (3) in the file path and file name and (4) in an accompanying json sidecar file. Hence, to be as versatile as possible, all four sources of information are represented in a BIDS mapping:

1. The file system metadata is contained in an input dictionary named “properties.” This dictionary contains file system properties of the data sample, i.e., the file path (in POSIX-style), the file name, the file size on disk, and the number of files in the containing folder. Depending on ones’ data management, this information allows or can help to identify the different data types in the source data repository.
2. The intrinsic metadata is contained in an input dictionary named “attributes.” This dictionary normally consists of a minimal subset of the available intrinsic metadata that is effective to identify the different data types in the source data repository.
3. The BIDS entities that define the file name after the conversion are contained in an output dictionary named “bids.”
4. The BIDS metadata is contained in an output dictionary named “meta.” The meta dictionary contains the custom key-value pairs that are added to a new or an existing json sidecar file by the bidscoiner plugins (further described later).

When source data is scanned by a BIDScoin routine, the keys of these input dictionaries indicate which metadata is to be extracted from the source data and matched against the dictionary value. In this identification procedure, the input dictionary values are interpreted by BIDScoin as regular expression patterns,^16^ and as such define the abovementioned broadness of the template or study bidsmap filters.

For instance, in a template bidsmap, a key-value pair of an attribute dictionary could be {ProtocolName: .*(mprage|T1w).*},^17^ which would extract the attribute string for “ProtocolName” from the DICOM^18^ header and tests if that string contains either a “mprage” or a “T1w” substring. BIDScoin will test all the key-value pairs of the input dictionaries and will consider it an overall match only if all of them tested positively. During the bidsmapper runtime, the existing attribute values are then replaced (expanded) by the full string values that were extracted from the header, e.g., {ProtocolName: t1_mprage_sag_p2_iso_1.0}, and then stored in the study bidsmap as a new BIDS mapping.

The expanded values will not contain broadly matching wildcards (.*) or Boolean “OR” operators (|), and hence act as very narrow regular expressions that make exact matches only. Note that the initial pattern from the template contains the prior knowledge that the data type is most likely “T1w” if the DICOM ProtocolName contains a “mprage” or “T1w” substring, but that the exact “t1_mprage_sag_p2_iso_1.0” substring is study-specific and cannot be predicted *a priori*. In this way, BIDScoin will collect all the source data types and will notify any unintended deviation from the data acquisition protocol.

**FIGURE 4:**
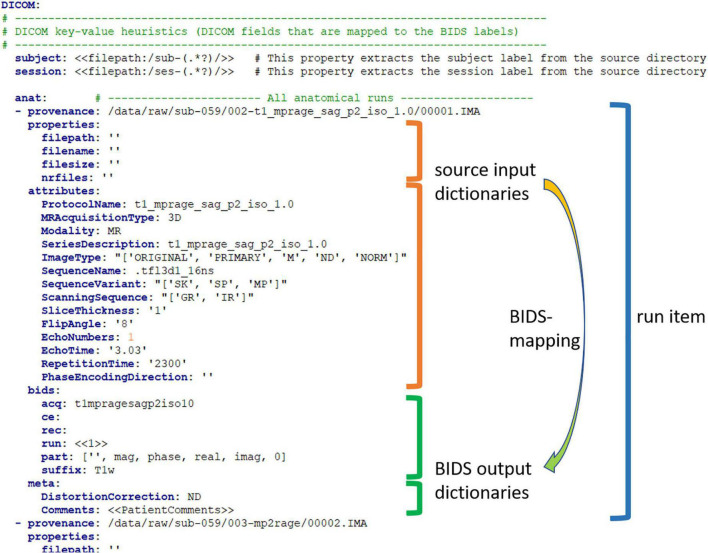
A snippet of study bidsmap in the YAML format. The bidsmap contains separate sections for each source data format (here “DICOM”) and subsections for the BIDS data types (here “anat”). The provenance field contains the pathname of a source data sample that is representative of the run-item. The provenance data is not strictly necessary but very useful for a deeper inspection of the source data and for back-tracing, e.g., in case of encountering unexpected results. The arrow illustrates how the “properties” and “attributes” input dictionaries are mapped onto the “bids” and “meta” output dictionaries. This BIDS mapping together with the provenance item, i.e., the run-item, is the fundamental building block of a bidsmap. Note that the “part” value in the bids dictionary is a list, which is presented in the bidseditor GUI as a drop-down menu (with the first empty item being selected). Also, note that the special double bracket dynamic values (<<1>> and <<PatientComments>>) are explained in section “Dynamic Values.”

Within a bidsmap, BIDS mappings are hierarchically grouped in BIDS data types, such as “anat,” “dwi,” and “func,” and accompanied with a “subject” and a “session” key-value pair for extracting BIDS subject and session labels. A snippet of a study bidsmap in the YAML^19^ format can be seen in Figure 4.

#### Dynamic Values

In the BIDScoin workflow, users can directly set the bidsmap values as they like, but often these values are already available as file attributes or properties or may vary between acquisitions. BIDScoin allows researchers to capture such cases with so-called “dynamic values.” Bidsmap values are treated as “dynamic” when they are captured between single (<>) or double brackets (<<>>), in which case the value should correspond to an attribute or a property key for which the value can be extracted from the data. Single brackets will always be extracted directly by BIDScoin routines and are typically part of a template bidsmap. Hence, when the template bidsmap is converted to a study bidsmap, the dynamic values are extracted and presented to the user for further editing. For instance, {acq: <ProtocolName>} in the bids output dictionary of the template bidsmap will appear as {acq: t1mpragesagp2iso10} in the study bidsmap (Figure 2). Double bracket dynamic values will remain as they are and will only be extracted during (bidscoiner) runtime, as explained further below.

Single bracket dynamic values are most useful as an intelligent first guess for the output dictionary values that vary only between data types, but not between acquisitions, such as the MRI sequence parameters “ProtocolName” or “FlipAngle.” Double bracket values can be useful for the dictionary values that vary more often, such as between subjects, sessions, or runs^20^ of the same data type. For instance, the value {Comments: <<PatientComments>>} in the meta dictionary (Figure 2) will extract the comments for that specific subject or session, while the value {subject: <<filepath:/sub-(.*?)/>>}^21^ will extract “003” (i.e., the shortest string between “/sub-” and “/”) if the data for that subject is in “/data/raw/sub-003/ses-01.” The latter example illustrates how a colon-separated regular expression can be appended to the “filepath” or “filename” property keys to extract a substring as researchers often encode multiple values or key-value pairs in a single filepath or filename. This mechanism to extract substrings is not limited to the file path or file name property keys but can be applied to any dynamic property or attribute key. For instance, {subject: <<PatientName:ID_(.*?)_>>} would likewise have extracted “003” if the DICOM attribute PatientName was, e.g., “ID_003_anon.” To test out dynamic values (either with or without appended regular expressions), users can handily enter them in the bidseditor within single brackets to instantly obtain their resulting value.

Dynamic values can handle many use cases and can be used throughout BIDScoin. Yet, two exceptions in the output dictionaries cannot always be handled directly with dynamic values. The first exception is the “run” index in the bids dictionary as this index cannot usually be determined from the data file alone. In that case, if the run-index is a dynamic number (e.g., {run: <<1>>}) and another output file with that run-index already exists, then during bidscoiner runtime this number will be incremented in compliance with BIDS standard (e.g., {run: 2}). If the run index is encoded in the header or file name, then the index can unambiguously be extracted using dynamic values. For instance, using {run: <<ProtolName:run-(.*?)_>>} will extract “3” if the DICOM ProtocolName is “t1_mprage_sag_run-3_iso_1.0.” The second exception not covered by dynamic values is the “IntendedFor”^22^ value in the meta dictionary, which also depends on the presence of other output files. Researchers can therefore specify IntendedFor images using a dynamic value with Unix shell-style wildcards. The bidscoiner will use these wildcards to lookup the appropriate images on disk. For instance, using {IntendedFor: <<task>>} will select all functional runs in the BIDS subject[/session] folder (as these runs always have “task” in their file name), and using {IntendedFor: <<Stop*Go><Reward>>} will select all “Stop1Go”-, “Stop2Go”-, and “Reward”-runs.

### The Plugin Interface for Interacting With Source Data

The BIDS community is working continuously to further improve and expand BIDS standard with new data types. Architecturally, to facilitate the implementation of such developments, all interactions of BIDScoin routines with the source data are done *via* a plugin layer that interacts in a data format-independent way. This paragraph describes the requirements and structure of plugins to allow advanced users and developers to write their own plugin and extend or customize BIDScoin to their needs. A BIDScoin plugin is a Python module with the following programming interface (functions):

- test(): A function to test the plugin and its options (see section “User Options”).
- is_sourcefile(): A function to assess whether a source file is supported by the plugin. The return value should correspond to a data format section in the bidsmap.
- get_attribute(): A function to read an attribute value from a source file.
- bidsmapper_plugin(): A function to discover BIDS mappings in a source data session. To avoid code duplications and minimize plugin development time, various support functions are available to the plugin programmer in BIDScoin’s library module named as “bids.”
- bidscoiner_plugin(): A function to convert a single source data session to bids according to the specified BIDS mappings. Various support functions are available in the “bids” library module.

Each plugin has its own section in a bidsmap to store and edit its discovered BIDS mappings. Plugins can be installed by the user but the plugins described in the following sections come pre-installed.

#### Dcm2niix2bids: A Plugin for DICOM and PAR/XML Data

The “dcm2niix2bids” plugin is a wrapper around the well-known pydicom (Mason et al., 2020), nibabel (Brett et al., 2020), and dcm2niix tools (Li et al., 2016) for interacting with and converting the DICOM and Philips PAR(/REC)/XML source data. Pydicom is used to read DICOM attributes, nibabel is used to read PAR/XML attribute values, and dcm2niix is used to convert the DICOM and PAR/XML source data to NIfTI^23^ and create BIDS sidecar files. These sidecar files contain standard metadata but, to give more control to the user, this metadata is appended or overwritten by the user data in the BIDS mapping meta dictionary. Dcm2niix2bids expects the source data files to be organized in:

• A “Series” subfolder organization. A Series folder is a subject[/session]^24^ -subfolder that contains files of a single data type, which are typically acquired in a single run – a.k.a “Series” in the DICOM standard. This format is often used by researchers in academic centers.
• A “DICOMDIR” organization with a DICOMDIR file in a single subject[/session] folder. A DICOMDIR is a dictionary file that indicates various places in a folder hierarchy of the available DICOM files. DICOMDIRs are often used in clinical settings.
• A flat DICOM organization. In a flat DICOM organization, all the DICOM files of all of the different Series are stored on a single subject[/session] folder. This organization is sometimes used when exporting data in clinical settings.
• A “PAR/XML” organization. All PAR/XML files of all the different Series in one folder. This organization is how users often export their data from Philips scanners in research settings (the session subfolder is optional): The PAR/XML session-data is expected to be organized in a single subject[/session] folder.

#### Spec2nii2bids: A Plugin for MR Spectroscopy Data

The “spec2nii2bids” plugin is a wrapper around the recent spec2nii^25^ Python library for interacting with and converting the MR spectroscopy source data. Presently, the spec2nii2bids plugin is the first implementation that supports the conversion to BIDS for Philips SPAR/SDAT files, Siemens Twix files, and GE P-files. As with the dcm2niix2bids plugin, the produced sidecar files already contain the standard metadata that is complemented or overruled by the metadata that users specified in the bidseditor. Also, spec2nii2bids expects the source data to be organized in subject[/session] folders.

#### Phys2bidscoin: A Plugin for Physiological Data

The “phys2bidscoin” plugin is a wrapper around the phys2bids Python library (The phys2bids developers et al., 2019) for interacting with and converting physiological source data. Phys2bids currently supports the conversion of labchart (ADInstruments, Sydney, Australia) and AcqKnowledge (BIOPAC, Goleta, CA, United States) source files to compressed tab-separated value (“.tsv.gz”) files and create their json sidecar files, as per BIDS specifications. As in the other plugins, the sidecar files contain the standard metadata that is overwritten by the user data entered in the bidseditor. Phys2bidscoin expects the source data files to be organized in subject[/session] folders. This plugin has been developed during the OHBM hackathon 2021 and is still considered experimental at the moment of writing.

### User Options

**FIGURE 5:**
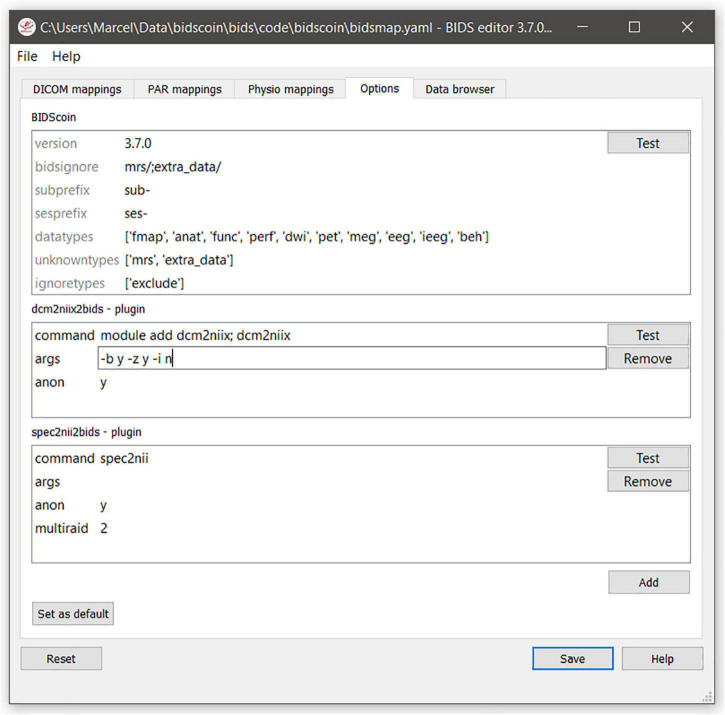
The bidsmap options for BIDScoin and its plugins. Note that how the GUI automatically adapts with a new “Physio” tab due to the presence of physiological data in the repository, i.e., a “Physio” section in the study bidsmap. The user can manage the plugins that will be used with the “Add” and “Remove” buttons, and save the current options to the template bidsmap using the “Set default” button.

A bidsmap contains a separate “Options” section with dictionaries for the options and settings of BIDScoin and its plugins. Only plugins that are listed in the Options will be used by the BIDScoin routines, which allow researchers to use different plugins for different data sets. The template bidsmap Options are taken as default and can be adjusted using the bidseditor (Figure 5).

#### The BIDScoin Options

• **version:** Used to check for version conflicts between the installed version and the original version in the bidsmap (e.g., when upgrading the software after creating the bidsmap) or between the installed version and the latest online version.
• **bidsignore:** A semicolon-separated list of non-BIDS data types that are added to the .bidsignore file.
• subprefix: The subject prefix of the source data, e.g., “sub-.”
• sesprefix: The session prefix of the source data, e.g., “ses-.”
• datatypes: The list of data types that are converted to BIDS.
• unknowntypes: The list of data types that are converted to BIDS-like data type folders.
• ignoretypes: The list of data types that are excluded/not converted to BIDS.

#### The Dcm2niix2bids Plugin Options

• **command:** The command to run dcm2niix on the user system, e.g., “module add dcm2niix; dcm2niix.”
• **args:** Argument string that is passed to dcm2niix to customize its behavior, e.g., −z n −i y for ignoring the derived data and having the uncompressed output data. The [Test] button can be used to test the proper working of the plugin.
• **anon:** Anonymization option (y/n) to round off age and discard acquisition date from the metadata.

#### The Spec2nii2bids Plugin Options

• command: The command to run spec2nii on the user system, e.g., “module add spec2nii; spec2nii.”
• args: Argument string that is passed to spec2nii to customize its behavior.
• anon: Anonymization option (y/n) to round off age and discard acquisition date from the metadata.
• multiraid: A spec2nii (mapVBVD) argument for selecting the multiraid Twix file to load (default 2).

#### The Phys2bidscoin Plugin Options

The options and settings of this plugin are still under development.

## Results

BIDScoin has been developed in the Donders Institute for Cognitive Neuroimaging at the Radboud University. A large number of researchers inside and outside this institute have successfully used BIDScoin to convert their data sets to BIDS, exposing it to a wide range of source data formats, data types, data organizations, experimental paradigms, and equipment manufacturers. In this paper, we present a full workflow using tutorial MRI data. The goal of this tutorial is to demonstrate BIDScoin’s functionality using the data that is representative of what researchers acquire in a standard neuroimaging experiment.

### Tutorial MRI Data

The following steps are part of a tutorial that allows users to download phantom MRI data, to test the complete BIDScoin workflow, and to compare it to a reference output. It is assumed that BIDScoin 3.7 is installed and that the path string for “dcm2niix” in the template bidsmap “Options” section has been set correctly (see section “The Dcm2niix2bids Plugin Options”).

#### Data Preparation

Before we can launch the GUI application and convert the data, we need to obtain a minimally organized source data set. In a shell terminal, create a tutorial playground folder by executing these commands:

$ bidscoin ––download .  # Download the tutorial data (use a “.” for the current folder or adapt it to your needs)

$ cd bidscointutorial  # Go to the downloaded data (or provide the path to the subfolders when calling the bidscoin tools)

The new “bidscointutorial” folder contains a “raw” source data folder and a “bids_ref” reference BIDS folder. The aim of this tutorial is to reproduce this bids_ref data folder. In the raw folder, these DICOM series (aka “runs”) will be found:

- **001-localizer_32ch-head**: A localizer scan that is not scientifically relevant and can be left out of the BIDS data set.
- **002-AAHead_Scout_32ch-head**: A localizer scan that is not scientifically relevant and can be left out of the BIDS data set.
- **007-t1_mprage_sag_ipat2_1p0iso**: An anatomical T1-weighted scan.
- **047-cmrr_2p4iso_mb8_TR0700_SBRef**: A single-band reference scan of the subsequent multi-band functional MRI scan.
- **048-cmrr_2p4iso_mb8_TR0700**: A multi-band functional MRI scan.
- **049-field_map_2p4iso**: The field-map magnitude images of the first and second echo. Set as “magnitude1,” bidscoiner will recognize the format. This field-map is intended for the previous functional MRI scan.
- **050-field_map_2p4iso**: The field-map phase difference image of the first and second echo.
- **059-cmrr_2p5iso_mb3me3_TR1500_SBRef**: A single-band reference scan of the subsequent multi-echo functional MRI scan.
- **060-cmrr_2p5iso_mb3me3_TR1500**: A multi-band multi-echo functional MRI scan.
- **061-field_map_2p5iso**: Idem, the field-map magnitude images of the first and second echo, intended for the previous functional MRI scan.
- **062-field_map_2p5iso**: Idem, the field-map phase difference image of the first and second echo.

Start with inspecting the raw data:

• Are the DICOM files for all the “bids/sub-*” folders organized in series-subfolders (e.g., “sub-001/ses-01/003-T1MPRAGE/0001.dcm,” etc.)? BIDScoin’s “dicomsort” utility can be used if this is not the case (hint: for didactical reasons this is not the case for sub-002). A help text for all BIDScoin tools is available by running the tool with the “-h” flag (e.g., “rawmapper -h”).
• The “rawmapper” utility can be used to print out the DICOM values of the “EchoTime,” “Sex,” and “AcquisitionDate” of the fMRI series in the “raw” folder.

#### Brain Imaging Data Structure Mapping

Now, a study bidsmap can be made, i.e., the mapping from DICOM source files to BIDS target files. To that end, scan all folders in the raw data collection by running this “bidsmapper” command:

$ bidsmapper raw bids

• In the GUI that appears at the end, edit the task and acquisition labels of the functional scans into something more readable, e.g., “task-Reward” for the “acq-mb8” scans and “task-Stop” for the “acq-mb3me3 scans.” Also, make the name of the T1 scan more user-friendly, e.g., by naming the acquisition label simply “acq-mprage.”
• Add a search pattern to the “IntendedFor” field such that the first field-map will select the “Reward” runs and the second field-map the “Stop” runs.
• Since for this data set, we only have one session per subject, remove the session label (and note how the output names simplify, omitting the session subfolders and labels).
• When all done, go to the “Options” tab and change the “dcm2niix” settings to get the uncompressed NIfTI output data (i.e., “*.nii” instead of “*.nii.gz”). Test the tool to see if it can run and, as a final step, save the study bidsmap. Close the editor and re-edit the study bidsmap by running: $ bidseditor bids. See what happens if you remove the compulsory task label of a functional scan or if you enter values in the output dictionaries that are not BIDS-compliant, such as non-alphabetic characters.

#### Brain Imaging Data Structure Coining

The next step, converting the source data into a BIDS collection, is straightforward and can be repeated whenever the new data has come in. To convert the data, run the “bidscoiner” command-line tool (note that the input is the same as for the bidsmapper):

$ bidsmapper raw bids

• Check the “bids/code/bidscoin/bidscoiner.log” file and note that it contains the complete terminal output. Check the “bids/code/bidscoin/bidscoiner.errors” file and see if any warnings or errors did occur.
• Compare the results in the “bids/sub-*” subject folders with the in “bids_ref” reference result. Are the file and folder names the same (ignore the multi-echo and “extra_data” images)? Also check the json sidecar files of the field-maps. Do they have the right “EchoTime” and “IntendedFor” fields?
• Re-run the bidscoiner command. Are the same subjects processed again? Forcefully re-run “sub-001.”

#### Finishing Up

Once the source data has been converted to BIDS, one still needs to do some additional work to make it ready for data analysis and sharing.

• Inspect the “participants.tsv” file and decide if it is ok.
• Update the “dataset_description.json” and “README” files.
• Combine the echoes using the “echocombine” tool, such that the individual echo images are replaced by the echo-combined image.
• Deface the anatomical scans using the “deface” tool. This will take a while but will obviously not work as normal for the (faceless) tutorial phantom data set. Therefore, store the “defaced” output in the “derivatives” folder (instead of, e.g., overwriting the existing images).
• As a final step, run the bids-validator^26^ on your “bids_tutorial” folder. Is the BIDS repository now ready to be shared?

## Discussion

Brain Imaging Data Structure standard is paving the way for more sharing of neuroimaging data that can efficiently be processed in a standardized manner. In this paper, we have demonstrated the use case for and main working of our flexible and user-friendly BIDScoin application that can convert a variety of raw neuroimaging data formats to the latest BIDS version 1.6. BIDScoin adopts an intelligent mapping strategy to discover and convert source data as opposed to using programmatic logic. BIDScoin is designed to make as much use as possible of the information available on disk, i.e., the file properties and attributes, as well as of the information that can be retrieved directly from the user.

An important part in the workflow is a step in which the user can edit the resulting output file names and add additional metadata. This, in itself, is not a trivial task as BIDS standard is ever increasing and many of its entities are not self-explanatory. The bidseditor GUI is therefore equipped with many help functions, such as help texts, tooltips, visual cues, informative pop-up windows, field input validations, reset buttons, and data inspection windows. In addition, users can consult the online documentation or, for instance, ask questions on the GitHub BIDScoin issue page. The user-friendliness of applications such as BIDScoin is important as it reduces the amount of non-scientific work in the scientific process and, hence, allows neuroscientists to devote more time and energy to address their research questions of interest.

### Advantages of BIDScoin

BIDScoin is a flexible framework for various reasons. It is written in Python and packaged and publicly released to pypi, hence the installation on multiple platforms is supported, including Linux, Windows, and macOS. Architecturally, BIDScoin makes the use of installable plugins, which increases the user-facing flexibility (e.g., to non-MRI data) and decreases the programmer-facing development costs. Researchers can modify or create their own plugins for specific data types without having to modify BIDScoin. As the entire framework is free and open-source, users are welcome and encouraged to contribute in this way.

Another feature contributing to BIDScoin’s flexibility is an option to use regular expressions in the bidsmap, which are well known for their powerful string-searching algorithms and usage in many programming languages. Nevertheless, researchers normally do not need to know about or interact with regular expressions as these are typically used in the template bidsmap and are already created by advanced users or developers.

Different researchers and research institutes use different data acquisition and management conventions. To accommodate for this, BIDScoin users can customize the data discovery intelligence in the default template bidsmap and reduce the number of edits they need to make in the GUI. Such customization can be as simple as changing the attribute or property strings to reflect their prior knowledge about the data – a task that does not require any programming knowledge from the user.

BIDScoin errors, warnings, and normal operations on the bidsmap or on the data are printed in a standard human readable format in the terminal and simultaneously stored in logfiles in the BIDS output folder. The study bidsmap itself, with all its mapping values and options, is also stored in the BIDS output folder. The provenance of the data and its conversion to BIDS are therefore always searchable, verifiable, and reproducible.

Converting source data to BIDS cannot always be done using a single application. BIDScoin only adds data and does not delete or overwrite the existing data (unless the user specifies so) and is therefore safe to use in conjunction with other BIDS applications.

### Limitations

While BIDScoin offers a convenient and flexible infrastructure for converting source data to BIDS, there are a few limitations to consider.

First, BIDScoin uses regular expressions instead of programmatic logic to map out source data. While this is a main feature, it also comes with the drawback that in certain situations the list of BIDS mappings in the bidsmap can become quite long and somewhat labor intensive to maintain or edit. This may become apparent when researchers have very irregular ways to acquire the data, such as manually entered file names that vary slightly. Moreover, in a mapping approach, it may be more difficult to deal with exceptional sessions in which certain runs need to be treated differently from others. In those situations, users may need to write a (small) plugin to solve such cases programmatically.

Second, BIDScoin has initially been developed with MRI data in mind. This means that the current support for other source data formats is not as mature as that of MRI, or not (yet) present. Researchers may therefore need to do post-processing with additional software to obtain a fully converted BIDS-compliant data set. A common example that is not handled by BIDScoin is the conversion to BIDS of stimulus presentation logfiles. This conversion is difficult to automate in a generic way as the logfiles typically vary between experimental paradigms and researchers.

Third, while the BIDScoin GUI provides an easy way for researchers to add their knowledge about the different data types to the BIDS output folder, it does not do so for the few modality agnostic files, such as the “dataset_description.json” file in the root of the BIDS folder. BIDScoin creates these files with placeholder content if they are not present already, but users still need to open these files with a text editor afterward to add content.

Finally, BIDScoin requires a minimally organized source data repository with a subject[/session] folder structure. Although this is very common practice, some researchers may have a different organization or use data management solutions such as PACS (Choplin, 1992), XNAT (Marcus et al., 2007), or DataLad (Halchenko et al., 2021). In those cases, researchers may need to export or reorganize their source data or write a custom plugin before they can use BIDScoin.

### Conclusion and Future Developments

BIDScoin is a new free and open-source framework for converting source data to BIDS. Its main features are flexibility and user-friendliness, that facilitate further adoption of BIDS standard, thus promoting data sharing and reproducibility. Currently, a plugin for physiological recordings is implemented and under testing, and a new PET plugin is under development. However, as the BIDS community and standard are continuously expanding, there is a need to develop more plugins to support more data formats and interface with data management solutions such as DataLad. With such a growing codebase in mind, it is important to grow a larger community of BIDScoin developers and to improve quality control by increasing the code coverage of the automated tests. An additional planned development is to release containerized versions of the software (Nichols et al., 2017) to deal with potentially increasingly complex dependencies and ensure exact reproducibility. At present, there is already a configuration file for Linux users to build their own BIDScoin Singularity container.

## Data Availability Statement

Publicly available datasets were analyzed in this study. This data can be found here: SURFdrive, https://surfdrive.surf.nl/files/index.php/s/HTxdUbykBZm2cYM/download.

## Author Contributions

All authors contributed to the manuscript and approved the submitted version. MZ was the creator, main designer, and main code contributor of BIDScoin, and main writer of the manuscript. SM contributed to the phys2bidscoin plugin code and was a co-writer of the manuscript. RO contributed to the design of BIDScoin and was a co-writer of this manuscript.

## Conflict of Interest

The authors declare that the research was conducted in the absence of any commercial or financial relationships that could be construed as a potential conflict of interest.

## Publisher’s Note

All claims expressed in this article are solely those of the authors and do not necessarily represent those of their affiliated organizations, or those of the publisher, the editors and the reviewers. Any product that may be evaluated in this article, or claim that may be made by its manufacturer, is not guaranteed or endorsed by the publisher.

## Footnotes

1 https://bids.neuroimaging.io/get_involved.html#extending-the-bids-specification.

2 https://bids-apps.neuroimaging.io.

3 https://bids.neuroimaging.io/benefits.html#converters.

4 https://github.com/cbedetti/Dcm2Bids.

5 https://github.com/jmtyszka/bidskit.

6 https://www.fieldtriptoolbox.org/reference/data2bids.

7 https://github.com/arnodelorme/bids-matlab-tools.

8 https://github.com/brainlife/ezbids.

9 https://github.com/mslw/horos-bids-output.

10 https://github.com/DrMichaelLindner/pyBIDSconv.

11 https://github.com/psychoinformatics-de/datalad-hirni.

12 https://github.com/Macquarie-MEG-Research/Biscuit.

13 https://github.com/Donders-Institute/bidscoin.

14 https://pypi.org/project/bidscoin.

15 https://bidscoin.readthedocs.io.

16 https://docs.python.org/3/library/re.html.

17 The Python syntax that is used throughout this paper of a key-value pair is {key: value}. Here, the .*(mprage|T1w).* value is taken as a regular expression pattern. In regular expressions, the “.*” in the pattern denotes a wildcard, the “|” denotes an OR statement, and the parenthesis indicate a grouping. The expression is evaluated in Python as: match = re.fullmatch(pattern, string).

18 http://dicom.nema.org.

19 http://yaml.org.

20 In BIDS, a run is defined as: an uninterrupted repetition of data acquisition that has the same acquisition parameters and task (however, events can change from run to run due to a different subject response or randomized nature of the stimuli). Run is a synonym of a data acquisition. Note that “uninterrupted” may look different by modality due to the nature of the recording. For example, in MRI or MEG, if a subject leaves the scanner, the acquisition must be restarted. For some types of PET acquisitions, a subject may leave and re-enter the scanner without interrupting the scan.

21 In regular expressions, the “.*?” pattern denotes a non-greedy wildcard. The substring that matches with the pattern between the parentheses is captured as the expression is evaluated in Python as: substring = re.findall(pattern, string).

22 IntendedFor values are used in BIDS to semantically indicate the association of an acquired data file with other acquired data files. A prominent example is a field-map that is acquired to correct fMRI images.

23 http://nifti.nimh.nih.gov.

24 Brackets indicate that this subfolder is optional.

25 https://github.com/wtclarke/spec2nii.

26 https://bids-standard.github.io/bids-validator.
